# Toward individualized dosimetry for radiopharmaceutical therapy in day-to-day clinical practice of nuclear oncology: overcoming heterogeneity of radiation-absorbed dose to tumor and critical organs

**DOI:** 10.1007/s00259-023-06420-z

**Published:** 2023-09-15

**Authors:** Lisa Bodei, R. Michael Tuttle, Ravinder K. Grewal, Audrey Mauguen, Finn Augensen, Murad Abusamra, Sonia Mahajan, Vetri Sudar Jayaprakasam, Joseph R. Osborne, Sofia Haque, Bernadette Z. Y. Wong, Ronald A. Ghossein, James Fagin, Heiko Schӧder, Alan Ho, John L. Humm, Steven M. Larson

**Affiliations:** 1 Department of Radiology, Memorial Sloan Kettering Cancer Center, 1275 York Ave, Box 77, New York, NY 2C-21210065 USA; 2 Department of Medicine, Memorial Sloan Kettering Cancer Center, New York, NY USA; 3 Department of Epidemiology & Biostatistics, Memorial Sloan Kettering Cancer Center, New York, NY USA; 4 Department of Medical Physics, Memorial Sloan Kettering Cancer Center, New York, NY USA; 5 Division of Molecular Imaging and Therapeutics, Weill Cornell Medical College, New York, NY USA; 6 Department of Pathology and Laboratory Medicine, Memorial Sloan Kettering Cancer Center, New York, NY USA

Radioactive iodine-131 (RAI) therapy of metastatic well-differentiated thyroid cancer (mDTC) has been a cornerstone of nuclear medicine practice since it was first conceptualized in the 1940s by Saul Hertz and Arthur Roberts. RAI continues to be an important part of the therapeutic armamentarium for advanced thyroid cancer in both adults and children.

## Practical RAI dosimetry as a clinical tool

In a recent article published in EJNMMI [1], we introduced an approach to define a PET imaging–based single-timepoint biomarker for estimation of radiation-absorbed dose (cGy) of mDTC RAI for advanced disease. Our methodology could guide the selection of the amount of radioactivity (MBq) needed to achieve a desired lesion dose (in cGy), based on standard uptake value (SUV) ^124^I at 48 h post-oral administration of 6 mCi Na[^124^I]I. The method employs a regression model relating the 48-h ^124^I lesion SUV measurements to the radiation-absorbed doses for these same lesions, and is illustrated on 208 individual lesions in 21 patients (our learning set). The model is derived from fitting a pharmacokinetic “gold-standard” radioactivity retention curve for each lesion based on four serial ^124^I PET images conducted at the nominal imaging times of 4, 24, 48, and 120 h post-radiotracer administration. If the performance of the approach is validated, the prescribing physician will be able to select an activity (MBq) to safely administer to patients with multiple metastatic lesions of heterogenous radioiodine avidity, to treat a desired fraction of the lesions to a specified radiation-absorbed dose.

The use of these results, such as those presented in both Fig. 2 and Table 3 from our aforementioned article [1], would facilitate treatment planning. Figure 2 contains data obtained by imaging the group of 21 mDTC patients to characterize individual lesion uptake and retention, to determine integrated retained radiation over time as recommended by the MIRD Committee. Table 1 shows, for the given SUVs at 48 h, the amount of radioactivity of ^131^I in GBq (mCi) estimated to produce at least 2000 cGy per lesion in 90%, 95%, and 97.5% of all lesions.

**Table 1:** Using the SUV at 48 h to guide recommendations for administered activity based on the likelihood that a fixed percent of metastatic lesions will receive a lesional dose >2000 cGy* (learning set)

SUV at 48 h	90%		95%		97.5%	
	GBq	(mCi)	GBq	(mCi)	GBq	(mCi)
15	10.8	(292)	13.2	(355)	15.4	(416)
20	8.1	(218)	9.4	(255)	11.4	(307)
30	5.7	(153)	6.6	(179)	7.5	(203)

*Based on prediction interval per mCi RAI administered; data from Table 3 in EJNMMI [1]

The individual lesion SUVs provide valuable information about intra-individual tumor heterogeneity, allowing clinicians to prescribe an administered activity that will optimize the therapeutic efficacy of RAI while bearing in mind the risks associated with RAI therapy. For example, if the vast majority of the clinically significant metastatic lesions demonstrate an SUV of 30 at 48 h and only one dominant lesion demonstrates an SUV of 15, we may choose to prescribe an administered activity of 7.5 GBq (203 mCi) to deliver >2000 cGy to most of the metastatic lesions with a plan for surgical resection or external beam irradiation of the dominant metastatic lesion—rather than administering 15.4 GBq (416 mCi), which would be required to achieve 2000 cGy in the dominant lesion. These decisions are made by a multidisciplinary team that develops multimodality treatment plans to achieve an optimal balance between therapeutic efficacy and risks, with the understanding that RAI can be used in concert with other treatment modalities for any individual patient.

At MSK, we routinely perform whole-body dosimetry on candidates for RAI therapy, to determine “maximum tolerated activity” (MTA), principal dose-limiting tissue being the blood (<200 cGy) [1]. The MTA offers a safety net to prevent excess toxicity (“*primum non nocere*”). But it is equally important to identify the minimum effective amount of radiation for tumor treatment despite heterogeneity of tumor uptake. In our RAI-treated mDTC population [1], among 15 patients, the ^124^I estimated cGy radiation-absorbed dose in 168 metastatic tumors was evaluated in relationship to the prescribed >2000 cGy therapy threshold. We determined that 96% of lesions received the predicted cGy radiation-absorbed dose >2000 cGy from the total amount of RAI administered, even though cGy heterogeneity varied over ~2 logs around the median lesion dose of ~22,000 cGy. Although currently our work is proof of principle, we find these initial observations encouraging enough to warrant effort to optimize our approach to make it suitable for clinical practice. We intend to (1) increase our learning set of patients (and thus the number of individual lesions) by approximately three-fold, and (2) obtain a validation set to evaluate effectiveness of treatment, including tumor size reduction with loss of ^131^I uptake, reduction of thyroglobulin, long-term survival, and, ultimately, cure.

## Radiopharmaceutical therapy expands to other solid tumors

While RAI therapy of mDTC is rightly considered the archetype, radiopharmaceutical therapy (RPT) is expanding rapidly to other solid tumors. In the last decade, new therapeutic radiopharmaceuticals and their associated treatment paradigms have been approved by regulatory agencies for advanced disease. These include Xofigo^®^ ([^223^Ra]RaCl_2_) and Pluvicto^®^ ([^177^Lu]Lu-PSMA-617) for prostate cancer and Lutathera^®^ ([^177^Lu]Lu-DOTATATE) for neuroendocrine tumors (NETs). Although the mechanisms of radionuclide tumor localization are unique to each therapy, we believe that all RPT methods are likely to feature wide heterogeneity of radiation-absorbed dose to the tumor’s targets. In this editorial, we propose that the theranostic approach applied to RAI could be applied to other heterogenous tumor-targeting agents such as [^177^Lu]Lu-PSMA-617 and [^177^Lu]Lu-DOTATATE.

## Targeted radiation, treatment response, and the need for dosimetry of RPT

It is our view that all RPT will benefit from both lesion-specific and patient-specific dosimetry: lesion-specific dosimetry because (a) growing evidence indicates that individual lesion response to RPT is directly related to dose (cGy) administered to individual lesions [2], and (b) inter- and intra-patient heterogeneity in lesion cGy may be profound regardless of tumor type and is intrinsic to the heterogeneous tumor features (target expression, interstitial pressure, oxygenation, etc.). The need for patient-specific dosimetry also comes from the individual variability in catabolism and clearance of therapeutic radiopharmaceuticals from normal tissues. Patient-specific dosimetry is particularly important when high-dose RPT (e.g., activity >100 mCi of ^131^I, or cumulative activity 22–44 GBq/600–1200 mCi of [^177^Lu]Lu-PSMA-617 or 22–29 GBq/600–800 mCi of [^177^Lu]Lu-DOTATATE) is to be administered.

## mDTC lesion radiation dose response

Because RAI has been practiced the longest, we have the most information about this form of targeted therapy. The common parameter for “tumor complete response” has been the disappearance of ^131^I uptake after treatment [3]. Using this criterion, Maxon used probe measurements during RAI and reported that 8500 cGy will provide a complete response for 75% of mDTC metastatic cervical lymph nodes, but 30,000 cGy is required to ablate residual thyroid disease [4]. Pioneering applications using the ^124^I/^131^I theranostic pair from Jentzen et al. extended knowledge of dosimetry in mDTC [5]. These studies not only confirmed the general conclusions of Maxon et al. with regard to dose response for normal thyroid remnants and mDTC in lymph nodes, but also reported that lesion dose response may vary depending on the location of disease, and that metastases to bone were difficult to eradicate, requiring cumulative doses of 35,000–60,000 cGy [2]. Durante et al. used sustained disappearance of ^131^I uptake as a criterion, noting long-term complete response in 43% of mDTC patients treated with 4×100 mCi of Na[^131^I]I every 6 months [3]. Furthermore, this group reported that once patients became negative for uptake, only 7% experienced subsequent tumor recurrence. Overall, 10-year survival after initial RAI was 92% in patients with sustained disappearance of ^131^I uptake and 19% in those whose uptake persisted. Thus, long-term progression-free survival has been achieved with ^131^I in a substantial proportion of patients.

## NET and prostate RPT—lesion and patient radiation dosimetry

Dosimetry estimates in relationship to tumor response and toxicity of normal tissues are beginning to appear for [^177^Lu]Lu-PSMA-617 and [^177^Lu]Lu-DOTATATE. For [^177^Lu]Lu-DOTATATE, as for other forms of RPT, wide inter- and intra-patient variability of tumor-absorbed doses has been reported for similar cumulative administered activities, making the dosimetry representation with the range more meaningful than with median values: doses may range from 1–2 to more than 5,000 cGy/GBq. It is clear that many tumors receiving less than 1000 cGy are gravely undertreated [6]. For many years, skepticism toward the predictive value of dosimetry prevailed, probably related to the limited understanding of tumor dose variability, scarcity of data correlating tumor dose with response, and lack of consideration of the role of radiosensitivity in radiation response [7]. More recently, prospectively acquired dose response curves have consistently shown a correlation between tumor-absorbed dose and lesion volume reduction assessed by CT, especially significant for lesions >4 cm when quantitative SPECT reconstruction with accurate partial volume corrections could be applied [8].

While the long-established skepticism has delayed the planning of large prospective randomized dosimetry trials to assess the advantages of dosimetry-based treatments, available dose response curves seem to indicate that, optimally, tumor doses of 12,000–13,000 cGy are necessary to effectively treat NETs [9]. Implementing faster dosimetry protocols (e.g., single- or double-point) will facilitate the practicality, acceptance, and inclusion of dosimetry as an integral part of treatment to verify that tumor doses fall within the expected therapeutic range and exposed dose-limiting tissues within the constraints for normal organ toxicity. In parallel, measurement of the radiation doses to tumor and normal organs will both improve our understanding of lesion radiosensitivity through the correlation with response [10] and help better define the dose-limiting toxicity to organs such as the kidney and salivary gland, resulting from RPT. Prostate cancer tumor radiation dose response to RPT has been reported by Hoffman and colleagues, using 50% reduction in circulating PSA as a response parameter. A median effective radiation-absorbed dose is approximately 1100 cGy, and these higher doses are correlated with a 50% drop in PSA, compared to those patients with no PSA decline (median ~900 cGy). Heterogeneity of 20- to 30-fold has also been reported for the variation in individual lesion cGy, with upper bounds not quite reaching 10,000 cGy [11]. Recently, the dosimetric results of the VISION trial sub-study were presented, indicating mean absorbed doses of 5.4 (range, 0.13–45) Gy/GBq for bone and 9.7 (0.99–55) Gy/GBq for nodes, in line with prior literature [12]. No dose response correlations have yet been presented, although one could infer that the higher absorbed doses correlate with more favorable radiological PFS, as reported in the main study [13].

For both [^177^Lu]Lu-DOTATATE and [^177^Lu]Lu-PSMA-617, kidney and bone marrow toxicity were seen in a small percentage of patients, which appeared to be the dose-limiting organs. To help with patient selection, a proposed criterion for pre-treatment with [^177^Lu]Lu-PSMA-617 has been a ratio of 1.5 for [^68^Ga]Ga-PSMA-11 SUV in tumor/liver, predicting beneficial response when [^68^Ga]Ga-PSMA-11 is used prior to [^177^Lu]Lu-PSMA-617 therapy.

## Common features of RPT in NaI-131 in thyroid cancer, [^177^Lu]Lu-PSMA-617 in prostate cancer, and [^177^Lu]Lu-DOTATATE in NETs

These targeted radionuclide therapies exhibit significant commonalities: namely, a variable number of lesions with highly heterogeneous uptake and retention, both between and within advanced patients with predominant clearance via the kidney. Toxicity depends on therapeutic index, i.e., the ratio of cGy tumor to cGy normal critical tissue. For these radionuclide therapies, dose-limiting toxicity is typically red marrow, but also potentially the kidney or, in rare instances, the lung. Knowing normal tissue dose limits for each RTP agent provides an opportunity for physicians to escalate the radiation-absorbed dose to tumor lesions so as to achieve the greatest efficacy and treatment response, avoiding serious radiation morbidities. Most current radionuclide therapies use a one-size-fits-all approach wherein all patients receive the same activity, without consideration for the patient’s tumor burden or pharmacokinetic profile of the RPT. At the other end of the spectrum, a highly personalized approach requires serial quantitative cross-sectional imaging (PET or SPECT) to perform dosimetry for all lesions and dose-limiting organs. Whereas this latter approach is preferred, few hospitals possess the resources to provide this level of care. Our proposed approach offers an intermediate solution based on a single 48-h pre-treatment PET scan with ^124^I, such as in the case of thyroid cancer. It is conceivable that the use of new theranostic pairs with imageable isotopes (e.g., ^89^Zr/^177^Lu, ^155^Tb/^161^Tb, ^203^Pb/^212^Pb, ^64^Cu/^68^Cu) will facilitate the expansion of this approach to other RPT strategies. Furthermore, the use of statistics on cohorts of patients can provide guidelines for the necessary amount of activity administered to achieve a target lesion dose with a defined probability. This approach is thus personalized based on the probability of successfully reaching the target treatment dose.

## Conclusion

Although requiring considerably more validation, we anticipate that the regression statistic approach for precision dosimetry (individualized per patient) that links the amount of radioactivity of a targeted radiopharmaceutical drug needed to be administered to a patient to achieve a therapeutic radiation dose threshold within a lesion population might be useful not only for RAI therapy of mDTC, but RPT for NETs and prostate solid tumors as well. Accordingly, we can begin to confront a major problem of current radionuclide targeted therapy: the wide heterogeneity of localization of *radiopharmaceutical to tumor* and the resulting unpredictable therapeutic index. Only through improved individual dosimetry can we tailor the radiation-absorbed dose to maximize efficacy of tumor treatment while minimizing normal organ toxicity. We call on our colleagues, experts in RPT, to consider joint action to implement true dosimetric approaches, including optimized biomarkers such as those we have proposed, to make individual lesion- and patient-specific organ RPT dosimetry practical for universal use.

## Data Availability

Not applicable
